# Geometry-Aware Abdominal Aortic Aneurysm Digital Twin for Patient-Specific Wall Stress Mapping

**DOI:** 10.21203/rs.3.rs-9971735/v1

**Published:** 2026-07-03

**Authors:** Julian Carvajal Rico, Victor De Oliveira, Satish C. Muluk, Mark K. Eskandari, Vikram S. Kashyap, Ender A. Finol

**Affiliations:** 1 Department of Mechanical, Aerospace, and Industrial Engineering, The University of Texas at San Antonio, San Antonio, TX 78249, USA; 2 Department of Statistics and Data Science, The University of Texas at San Antonio, San Antonio, TX 78249, USA; 3 Department of Thoracic and Cardiovascular Surgery, Allegheny Health Network, Allegheny General Hospital, Pittsburgh, PA 15212, USA; 4 Feinberg School of Medicine, Northwestern University, Chicago, IL 60611, USA; 5 Cardiovascular Health, Corewell Health, Grand Rapids, MI 49503, USA

**Keywords:** Abdominal Aortic Aneurysm, Wall Stress, Deep Learning, Graph Neural Networks, Transformers

## Abstract

Accurate estimation of wall stress distributions in abdominal aortic aneurysms (AAAs) is critical for improving rupture risk prediction beyond the traditional maximum diameter criterion. While high-fidelity finite element analysis (FEA) provides precise patient-specific wall stress estimates, its computational cost limits its use in real-time clinical decision making. We introduce a graph-based deep learning framework that leverages three types of graph neural networks (GNNs), namely a Gated Graph Convolutional Network (GGCN), an Equivariant Graph Neural Network (EGNN), and a Graph Transformer (GT) to rapidly predict FEA-derived wall stress distributions directly from AAA outer wall surface meshes. Computed tomography angiography (CTA) images from 202 AAA patients treated at three clinical centers, were segmented using an in-house U-Net pipeline and refined manually to generate high quality AAA volume and surface meshes. From these, several node-specific geometric and biomechanical features were recorded, including wall thickness (WallTHK), intraluminal thrombus thickness (ILTTHK), wall strength $\left(\sigma_{\text{ult}}\right)$, the principal minor and major curvatures ($k_{1}$ and $k_{2}$) and their derived curvatures (Gaussian K and mean M), distance to the lumen centerline ($r_{L}$), normalized local diameter (NORD), and the ILTTHK-to-WallTHK ratio ($\rho^{\text{ILT}}$). Local information was incorporated via neighborhood averaged features from the six nearest nodes. All GNNs achieved high fidelity in reproducing patient-specific FEA wall stress fields, with the GT providing the best overall agreement, while the GGCN and EGNN also delivered strong performances. The best performing GT captured spatial stress patterns, preserved biomechanical trends, and enabled near real-time inference. It reproduced FEA-derived wall stress with high accuracy (node-specific $R^{2}>0.97$, graph-level $R^{2}>0.98$) while reducing per-case inference time from hours for a complete FEA simulation to a few seconds on a single GPU. With further refinement and larger cohorts, this graph-based framework may strengthen rupture risk assessment and facilitate routine, patient-specific biomechanical analyses in clinical workflows.

## 1 Introduction

Abdominal aortic aneurysm (AAA) is a life-threatening vascular disease characterized by a permanent, localized dilation of the abdominal aorta. Although screening and surveillance have reduced the incidence of fatal ruptures, clinical decision making is still mainly based on the size of the aneurysm, in particular the maximum diameter and growth rate, to recommend elective repair^1^. In line with this practice, current medical guidelines recommend elective intervention at diameter thresholds of approximately ≥ 5.5 cm in men and 5.0 – 5.4 cm in women, with additional considerations for rapid expansion or symptoms^2, 3^. However, small aneurysms can rupture, for example, at a rate of ~23% for AAAs between 4.1–5.0 cm^4^. The clinical management criteria are convenient, but only indirectly reflect the underlying biomechanics that precipitate rupture. The risk of rupture is governed by the balance between local wall stress and wall strength; regions of complex geometry, heterogeneous wall thickness, and intraluminal thrombus (ILT) can create highly nonuniform stress fields that diameter alone cannot identify^1, 5, 6^.

FEA enables patient-specific estimation of wall stress, which has been repeatedly associated with improved risk stratification compared to diameter thresholds^7–9^. However, traditional FEA requires laborious preprocessing (segmentation, mesh generation, material model and wall thickness assumptions, specification of boundary conditions) and substantial computational time, which limits its use in routine practice^10^. In addition, uncertainty about material properties, the constitutive behavior of ILT, and boundary conditions can propagate to stress predictions, complicating clinical translation^6, 11–16^. Despite decades of research progress, biomechanical stress metrics have not been adopted for routine decision making in current clinical guidelines, and recent evidence highlights limited and heterogeneous data supporting their incremental value^3, 17^.

Advancements in machine learning (ML) and computer vision offer a complementary path: learn predictive mappings from image-derived anatomy to biomechanical stress directly from data. Robust automated segmentation with U-Net–style architectures has improved performance in reconstructing the AAA wall, lumen, and ILT^18–20^. Surface geometry encodes rich biomechanical information; features such as principal curvatures (minor $k_{1}$ and major $k_{2}$), mean (M) and Gaussian (K) curvatures, wall thickness (WallTHK), ILT thickness (ILTTHK), and centerline distance ($r_{L}$) correlate with wall stress concentrations and rupture-prone regions^21–25^. Because these descriptors are calculated on irregular vessel surfaces, graph neural networks (GNNs) provide a natural modeling framework by operating directly on the surface meshes as graphs, where message passing aggregates local and global geometric information^26–29^.

Faithfully capturing local interactions on complex aneurysm geometries remains a challenge. Standard convolutional or purely local GNNs may struggle to model distant but biomechanically coupled regions (e.g., across the sac or near the neck), while models that rely heavily on absolute coordinates can be sensitive to orientation and scale. In addition, clinically significant high stress nodes are rare relative to low-stress nodes; therefore, loss functions and readouts that emphasize tail behavior are needed to avoid systematic underestimation of extremes. Recent GNN architectures such as gated graph convolutional networks (GGCNs), equivariant graph neural networks (EGNNs), and Graph Transformers (GTs) provide complementary strategies to address these limitations by strengthening message passing, enforcing geometric symmetries, and enabling global and local attention over complex vessel wall meshes. Within this family of GNNs, we first consider a non-equivariant GGCN inspired by Gated Graph Sequence Neural Networks^30^. In this architecture, information is propagated along the wall mesh via gated message passing, where recurrent units (e.g., GRU-style updates) regulate how newly aggregated neighborhood information is integrated with existing node states. This gating mechanism helps stabilize optimization on large, irregular graphs and mitigates the over-smoothing and vanishing-gradient issues that can arise in deeper message-passing networks. For AAA modeling, the GGCN provides an expressive yet relatively lightweight baseline for modeling local and mid-range wall stress patterns, with a graph-based formulation that can be applied inductively to patient-specific surface meshes of varying resolution.

EGNNs complement the baseline by explicitly encoding the underlying Euclidean symmetries of the geometry. By constraining the network to be equivariant to translations and rotations, EGNN-type architectures can reuse information more effectively for differently oriented aneurysms (e.g. due to CT scanner orientation or patient positioning) and reduce the reliance on arbitrary coordinate frames^31–33^. This is particularly desirable when wall stress depends on shape and relative configuration rather than global positioning and when training data are limited or heterogeneous between imaging centers. In practice, this symmetry-aware inductive bias allows EGNNs to achieve strong performance with relatively modest capacity, making them a computationally efficient alternative to more complex attention-based architectures in 3D medical imaging settings. GTs extend self-attention to irregular domains by conditioning attention on graph structure and geometry, enabling information flow across distant but biomechanically coupled regions. Relative and positional encodings are key to robustness and long-range reasoning in attention^34^, while graph-specialized Transformer designs (e.g., spectral/Laplacian positional encodings and edge-aware keys/values) have shown strong performance in non-Euclidean domains^35, 36^. Moreover, recent GTs that incorporate distance and shortest-path signals achieve state-of-the-art accuracy on structural benchmarks^37^. For applications where strict orientation consistency is critical beyond the present work, equivariant attention architectures provide an alternative route, preserving prediction behavior under rigid motion while retaining the benefits of attention^38^. For AAA modeling, this attention-based formulation provides global receptive fields for modeling sac-to-neck and sac-to-branch interactions, enables orientation-insensitive reasoning through relative encodings, and remains compatible with large graphs via sparse attention over the mesh connectivity.

Informed by the aforementioned literature gaps, we view each patient-specific reconstruction as a geometry-aware *AAA digital twin* and seek fast, physically informed surrogates that map from surface meshes generated from computed tomography angiography (CTA) images to wall stress endpoints. Rather than treating peak, mean, and full-field stresses as separate modeling stages, we train node-specific surrogates that recover the spatial stress field on the aneurysm wall surface and then derive clinically salient global summaries, such as the mean wall stress $\left(\overset{‾}{\sigma }\right)$ and the 99^th^ percentile wall stress $\left(\sigma_{99}\right)$^7, 8^. With this formulation, an accurate reproduction of every nodal value is not required. Rather, the models are optimized to adequately reproduce the general stress distribution and high stress tail so that $\overset{‾}{\sigma }$ and $\sigma_{99}$ calculated from the predicted field closely match their FEA-estimated counterparts, supporting the assessment of individual rupture risk.

The present work describes a reproducible AAA wall stress *digital twin* pipeline and systematically evaluates three GNN architectures for graph- and node-specific stress targets by contributing:

1. A geometry *digital twin* pipeline that converts CTA images into standardized graph representations of the AAA outer wall with node-specific geometric and biomechanical descriptors, and spatial location.
2. A systematic benchmark of GGCN, EGNN, and GT models in an AAA cohort and preprocessing, clarifying which graph paradigms are most suited to estimate $\overset{‾}{\sigma },\sigma_{99}$, and full-field wall stress endpoints in this cohort.
3. Tail-aware training and readout strategies that enhance sensitivity to clinically important high stress regions without sacrificing overall accuracy.
4. A comprehensive evaluation on a multi-institution cohort of AAA CTA–derived geometries, reporting graph-level errors for $\overset{‾}{\sigma }$ and $\sigma_{99}$, node-specific accuracy and spatial correlations, and agreement of predicted vs. ground-truth FEA wall stress maps.

Framing wall stress estimation as a geometry-aware *digital twin* enables fast patient-specific wall stress mapping once the models are trained, improving scalability for large cohorts and longitudinal AAA surveillance. The same framework could be applied to other vascular pathologies that require biomechanical estimates and where the vasculature can be represented by a surface mesh with rapid, data-driven surrogates needed to complement traditional finite element modeling workflows. The remainder of the paper is organized as follows. Section 2 describes the generation of the data set and the computation of the features. Section 3 introduces graph-based surrogate models, including graph representation, model families, and training procedures. Section 4 presents quantitative and qualitative results for node-specific and graph-level wall stress endpoints. Section 5 discusses the clinical implications, limitations, and future directions for integrating *digital twin* wall stress mapping into clinical workflows, and Section 6 presents the conclusions.

## 2 Methods

**Figure 1.**
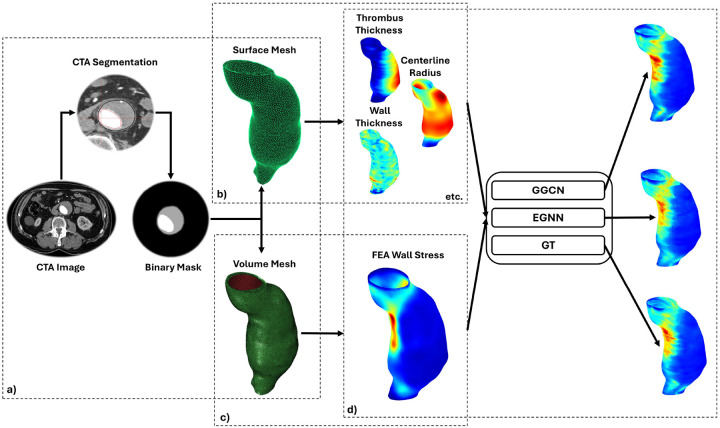
Geometry-aware *digital twin* framework for AAA wall stress mapping. (a) Segmentation from CTA imaging to binary masks, followed by reconstruction of surface and volume meshes. (b) Feature engineering on the standardized outer wall surface (e.g., WallTHK, ILTTHK, and $r_{L}$). (c) Biomechanical ground truth: first principal wall stress computed with the volume mesh via FEA and projected onto the outer wall surface. (d) Graph-based prediction of wall stress using GGCN, EGNN, and GT.

We propose a geometry-aware *digital twin* workflow that links CTA-derived anatomy to patient-specific wall stress endpoints. As shown schematically in Figure 1, the pipeline integrates: (i) image segmentation to obtain binary masks for the wall, ILT, and lumen; (ii) standardized surface reconstruction for feature computations; (iii) volumetric meshing and FEA for biomechanical ground truth; and (iv) supervised graph learning for wall stress prediction. From each CTA exam, an in-house U-Net code with expert refinement produces masks that serve two functions. First, to reconstruct the outer wall surface, which we standardize its resolution for all patients, and compute node-specific geometric/biomechanical descriptors, including WallTHK, ILTTHK, $k_{1},k_{2}$, M, K, $r_{L}$, the ILTTHK-to-WallTHK ratio ($\rho^{\text{ILT}}$), wall strength ($\sigma_{\text{ult}}$), and mesh coordinates (x,y,z) relative to the centroid (0,0,0) of the distal end of each AAA surface mesh. Second, to generate a volume mesh and compute first principal wall stresses in Abaqus (Dassault Systèmes, Waltham, MA), projecting the results back to the outer wall surface as the ground truth. We represent every AAA in the cohort as a surface graph whose nodes carry geometric/biomechanical features and wall stress targets, and we train three complementary GNN families: a GGCN, an EGNN, and a GT to estimate $\overset{‾}{\sigma },\sigma_{99}$, and the full wall stress field. Training uses standardized features, early stopping, and tail-aware objectives while evaluation reports graph- and node-specific errors and spatial agreement with the ground truth FEA output as the reference.

### 2.1 Cohort, Imaging, Segmentation, and Surface Standardization

We retrospectively acquired CTA exams from 202 AAA patients under surveillance following IRB-approved protocols at three clinical centers (Allegheny General Hospital in Pittsburgh, PA; Northwestern Memorial Hospital in Chicago, IL; Corewell Health in Grand Rapids, MI). From each exam, we used images of the abdominal aorta starting immediately below the left renal artery and ending at the aorto-iliac bifurcation. Data set acquisition was conducted in accordance with the Declaration of Helsinki, and informed consent was waived because of the retrospective design and use of existing de-identified clinical data.

The CTA images were processed with an in-house U-Net based algorithm^20^ that identified the outer wall, inner wall, and lumen boundaries, followed by expert refinement to correct artifacts and ensure anatomical fidelity, yielding four-region binary masks per CTA exam. (Figure 1.a). From the refined masks, we reconstructed triangulated surfaces for the outer wall and lumen using standard smoothing routines, preserving geometric detail relevant for wall stress calculation. The binary masks also served as input to the meshing pipeline that generated the surface and volume meshes used in the subsequent modeling calculations.

To make graph statistics comparable between patients and to allow for consistent batching, we standardized the outer wall surface resolution to a target of ≈ 150,000 nodes. The target size was derived by averaging node counts among AAAs with maximum diameter ≤ 7.7 cm, which avoids anchoring to extremes. We performed isotropic, curvature-preserving remeshing using a PyMeshLab-based wrapper, with a short calibration pass to estimate the relation between face count and edge length, followed by a final pass with up to two bounded refinements to reach a tolerance of ±2% in node count relative to the target. For each AAA we registered pre- and post-remeshing node and face counts, calibration settings, final error to target, number of refinement iterations, and key clinical metadata such as $\overset{‾}{\sigma }$ and $\sigma_{99}$, serving as quality control records. Visual inspection confirmed preservation of sac morphology, neck geometry, and local bulges, while removing redundant facets that can destabilize feature estimates and message passing.

### 2.2 Geometric and Biomechanical Descriptors

From the standardized outer wall, inner wall, and lumen surfaces, we computed node-specific descriptors that represent thicknesses, thrombus burden, surface shape, and position. These quantities summarize geometric measures that modulate wall stress and the likelihood of focal stress amplification. For each outer wall vertex at position $x_{i}$, its wall thickness is calculated as the minimum Euclidean distance to the inner wall surface vertex at position $y_{i}$, as given by Eq. (1),

(1) $${\text{WallTHK}}_{i}=\underset{y\in S_{\text{inner}}}{\text{min}}{\left\|x_{i}-y\right\|}_{2}.$$

ILTTHK is calculated as the minimum Euclidean distance from the outer wall vertex at position $x_{i}$ to the lumen surface vertex at position $z_{i}$, calculated using Eq. (2), providing a surrogate for local thrombus burden.


(2) $${\text{ILTTHK}}_{i}=\underset{z\in S_{\text{lumen}}}{\text{min}}{\left\|x_{i}-z\right\|}_{2}.$$

The principal curvatures $k_{1,i}\geq k_{2,i}$ are the eigenvalues of the shape operator at the surface point $x_{i}$, in the tangent plane, quantifying maximal and minimal normal bending along orthogonal directions^39, 40^. From these, K and M are defined by Eqs. (3) and (4). A positive K indicates dome-like regions; a negative K indicates saddle-shaped regions. Curvatures were estimated with a modified biquintic finite element (BQFE)^41^ patch fit to reduce discretization noise.

(3) $$K_{i}=k_{1,i}*k_{2,i}$$

(4) $$M_{i}=\frac{1}{2}\left(k_{1,i}+k_{2,i}\right).$$

$r_{L}$ encodes local caliber and sac-to-neck variation. It is calculated as the Euclidean distance from each outer wall vertex at position $x_{i}$ to the anatomically derived aortic centerline c(s), obtained by lumen skeletonization and smoothing^42, 43^, using Eq. (5).

(5) $$r_{L,i}=\underset{s}{\text{min}}{\left\|x_{i}-c(s)\right\|}_{2}.$$

$\rho^{\text{ILT}}$ represents the local thrombus burden relative to wall thickness and is used as a dimensionless indicator with a small ε for numerical stability, calculated using Eq. (6).


(6) $$\rho_{i}^{\text{ILT}}=\frac{{\text{ILTTHK}}_{i}}{{\text{WallTHK}}_{i}+\varepsilon }.$$

A wall strength surrogate was calculated at each node using a regression model^44^ that relates local thrombus burden, normalized diameter, aneurysm familial history, and sex to the ultimate wall strength. For node i, the wall strength is calculated in units of N/cm^2^ using Eq. (7).

(7) $$\sigma_{\text{ult},i}=72.9-33.5\left(\sqrt{{\text{ILT}}_{i}}-0.79\right)-12.3\left({\text{NORD}}_{i}-2.31\right)-24\text{HIST}+15\text{SEX},$$

where ${\text{ILT}}_{i}$ is the local ILTTHK (in cm), ${\text{NORD}}_{i}$ is the normalized local diameter (in cm), HIST encodes the AAA family history (+0.5 for positive history and −0.5 otherwise), and SEX encodes sex as +0.5 for male or −0.5 for female patients.

The spatial coordinates (x,y,z) are centered and scaled in a common patient space. During learning, models use standardized coordinates or relative encodings such as Δx, pairwise distances, or low-band Fourier features to promote invariance to translation and rotation.

### 2.3 Biomechanical Ground Truth (FEA)

Patient-specific wall stresses were computed from a lumen–thrombus–wall assembly using a standardized finite-element workflow that constructs a continuous ILT between the luminal and outer wall boundaries. From the refined binary masks, the outer wall and lumen surfaces were first meshed independently in AAAMesh^45^ to generate NASTRAN shell meshes with nominal constant wall thickness and case-specific in-plane resolution; the outer wall was also exported as an STL surface for further processing. The lumen and inner wall boundaries were then used to derive a continuous ILT volume, smoothing and remeshing the resulting volume.

**Figure 2.**
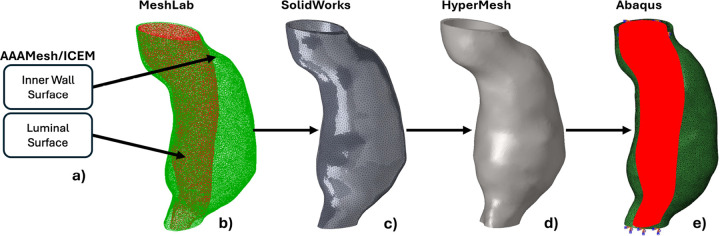
Schematic of the pipeline used to generate wall-ILT volume meshes for biomechanical analyses. (a) Extraction of the lumen and inner wall surfaces from AAAMesh^45^ and Ansys ICEM CFD; (b) Curvature-preserving remeshing and smoothing in MeshLab; (c) Conversion to a closed solid in SolidWorks; (d) Tetrahedral volume meshing in HyperMesh; and (e) Wall–ILT assembly with luminal surface imported into Abaqus for FEA simulation.

The lumen–thrombus–wall assembly was created with a multi-tool pipeline to ensure geometric continuity and mesh quality (Figure 2). The lumen and inner wall surfaces extracted from AAAMesh were subjected to a series of meshing and smoothing operations through Ansys ICEM CFD (Ansys Inc., Canonsburg, PA), MeshLab (Visual Computing Lab, ISTI-CNR, Italy) SolidWorks (Dassault Systèmes, Waltham, MA), and HyperMesh (Altair Engineering Inc., Frisco, TX). With the latter application, surface triangles were isotropically refined and converted to high-quality tetrahedrals (target ≈ 250,000–500,000 elements).

For the FEA simulations, the AAA wall was modeled as an isotropic hyperelastic Mooney–Rivlin material (reduced polynomial, order 2) with coefficients $C_{10}=17.4\;\text{N}/{\text{cm}}^{2}$ and $C_{20}=188.1\;\text{N}/{\text{cm}}^{2}$^6^, and the ILT was modeled as an isotropic Mooney–Rivlin material with $C_{10}=7.98\;\text{N}/{\text{cm}}^{2}$ and $C_{20}=8.71\;\text{N}/{\text{cm}}^{2}$^46^. A surface tie constraint was applied to the ILT-wall interface to prevent relative sliding. An mean intraluminal pressure of 93.33 mmHg was uniformly applied to the inner ILT and inner wall surfaces, while the proximal and distal ends were constrained to suppress rigid-body motion. Hybrid solid elements (C3D4H) were used for the ILT and 20-node hybrid brick elements (C3D20H) for the wall. Geometric nonlinearity was enabled and automatic time increments were used for quasi-static equilibrium.

After convergence, the first principal stress at the wall integration points was exported and mapped to the standardized outer wall surface mesh used for graph construction. Node-specific stresses ${\left\{\sigma_{i}\right\}}_{i=1}^{N}$ were obtained by nearest-point projection from surface nodes to the underlying volumetric elements, with a distance criterion to exclude ill-mapped nodes from subsequent statistics. From this mapped field, we retained node-specific stresses as supervision targets for the GNNs and derived graph-level scalars used in the evaluation, in particular the empirical 99^th^ percentile wall stress as given by Eq. (8),

(8) $$\sigma_{99}=\text{inf}\left\{s\in R:\frac{1}{N}\sum_{i=1}^{N}1\left\{\sigma_{i}\leq s\right\}\geq 0.99\right\},$$

which serves as a robust surrogate for peak wall loading. $\overset{‾}{\sigma }$ and $\sigma_{99}$ provide the biomechanical ground truth for training and evaluating node-specific wall stress maps and their clinically relevant global summaries.

## 3 Graph-Based Surrogate Models

To leverage GNNs for wall stress prediction, we view each AAA surface as a graph, so that local geometry and tissue properties become node-specific features, and the model can learn wall stress directly on the native surface mesh. This representation preserves the mesh connectivity while enabling message passing between neighboring wall regions.

### 3.1 Graph Representation

**Figure 3.**
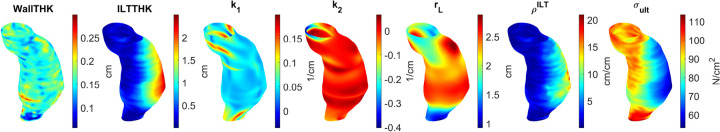
Exemplary graph representation of a patient-specific AAA. Color maps show selected node-specific geometric or biomechanical descriptors on the outer wall surface, illustrating the spatial variability of the features used as input to the GNNs.

Each AAA geometry is represented as a graph 𝒢=(𝒱,ℰ), where 𝒱 is the set of vertices (graph nodes) of the standardized outer wall surface (one node per mesh vertex) and ℰ is the set of undirected edges inherited from the surface mesh connectivity. The node feature vector $h_{i}$ for vertex i comprises the geometric descriptors detailed in Section 2.2, i.e. WallTHK, ILTTHK, $k_{1},k_{2}$, M, K, $r_{L},\rho^{\text{ILT}}$, and the spatial coordinates (x,y,z). To model short-range information strongly correlated with local wall stress, we augment these features with statistics calculated with the six nearest neighbors (by Euclidean distance). Specifically, for ILTTHK, $\rho^{\text{ILT}},k_{1},k_{2},\sigma_{\text{ult}}$, and $r_{L}$, we compute the mean, standard deviation, maximum, and delta (max–min), generating descriptors based on the six closest spatial neighbors (kNN-6), e.g. ${\text{ILTTHK}}_{\text{mean}}^{\text{knn}6},{k_{1}^{\text{kNN}-6}}_{\text{std}}$, and ${\text{r}}_{{\text{L}}_{\text{delta}}}^{\text{kNN}-6}$. Together with a categorical ILT class and simple products (e.g. $k_{1}$ × WallTHK), the augmentation yields 43 node-specific features per 𝒱 in the processed data set, some of which are illustrated for an exemplary AAA in Figure 3.

Because raw meshes exhibited large size variability (from 78,000 to 900,000 nodes), we first remeshed the outer wall surface of each AAA to a common target size of approximately 150,000 nodes, as described in Section 2.1. For the final cohort, the graphs contained 147,662–153,564 nodes (a mean ≈ 150,000 nodes) and 221,303–230,147 edges, ensuring comparable graph sizes for batching and training. In addition, we attached FEA-derived wall stresses as supervision signals. Each node i carries its mapped wall stress $\sigma_{i}$ as a scalar regression target, and we computed graph-level summaries directly from the predicted and reference fields, e.g., $\overset{‾}{\sigma }$ and $\sigma_{99}$. Global descriptors were stored as graph attributes and broadcast to nodes when used as inputs; these include maximum diameter (*MaxDia*, in cm), encoded SEX and ILT burden metrics ($\overset{‾}{\sigma },\sigma_{99}$, maximum $\rho^{\text{ILT}}$) together with an ILT class label. The three types of models (GGCN, EGNN, and GT) are based on the same node-specific feature vectors and mesh connectivity; EGNN also uses standardized coordinates for distance-based messages, while GT can be augmented with relative encodings derived from (x,y,z) to condition attention on geometric relationships throughout the aneurysm surface.

### 3.2 Model Types and Learning Tasks

We benchmarked three GNN architectures that share a common input–output formulation but differ in how they aggregate geometric information on the AAA wall. All models operate on the standardized outer wall graphs, use node-specific geometric descriptors, graph-level clinical attributes, and spatial coordinates as input features. They also predict the full node-specific wall stress field, from which $\overset{‾}{\sigma }$ and $\sigma_{99}$ are derived. The first architecture is a residual GGCN that serves as a strong, scalable baseline for irregular meshes. The second is an EGNN that explicitly encodes invariance to rigid motions via distance-based messages and coordinate updates. The third is a local GT that replaces conventional message passing with multi-head self-attention over the mesh connectivity to better replicate anisotropic and long-range geometric interactions.

#### Shared training setup.

For all architectures, node-specific inputs are formed by z-scoring and concatenating geometric descriptors, graph-level clinical attributes, and standardized coordinates, while normalized FEA wall stresses serve as regression targets. Training uses a composite, tail-aware loss that combines (i) a node-specific, stress-weighted mean squared error (up-weighting nodes in the upper stress quantiles), (ii) penalties on the discrepancy between predicted and true $\overset{‾}{\sigma }$ and between predicted and true $\sigma_{99}$, and (iii) a pinball (quantile) term to emphasize the high stress tail. To improve robustness and efficiency, we applied stress-stratified node subsampling within graphs, graph-level data augmentation on geometric and clinical descriptors, and a focal-style reweighting of “complex” aneurysms identified by high batch losses. All models were optimized with Adam and early stopping was applied as needed.

#### Gated graph convolution network (GGCN).

The GGCN operates directly on the standardized outer wall graphs for node-specific wall stress prediction. A linear projection first maps the input features to a hidden dimension, followed by a stack of gated graph convolution layers with layer normalization, rectified linear unit (ReLU) activations, dropout and residual connections, and a final linear readout outputs one scalar stress value per node (see section A.1 in the Supplementary Material).

#### Equivariant graph neural network (EGNN).

To explicitly encode geometric invariances, the EGNN jointly updates node features and coordinates using distance-based messages. Each layer computes edge messages from pairs of node features and squared inter-node distances, aggregates them at receiving nodes, and then applies (i) a node multilayer perceptron (MLP) to update features and (ii) a coordinate MLP to produce scalar edge-wise coefficients that drive equivariant coordinate updates along relative directions. Feature updates are followed by layer normalization, ReLU activations, dropout and residual connections, and a final linear readout maps hidden features to a single stress value per node (see section A.2 in the Supplementary Material).

#### Graph Transformer (GT).

The GT is built from stacked multi-head self-attention layers operating on the AAA wall mesh. Each GT layer applies attention restricted to the mesh adjacency, producing edge-aware updates that reweigh information from neighboring nodes before layer normalization, ReLU activations, dropout and residual connections, and a final linear layer maps hidden features to a scalar stress prediction per node. EGNN and GT thus provide complementary alternatives to GGCN by enforcing geometric symmetries and enabling flexible attention-based aggregation of wall geometry, respectively (see section A.3 in the Supplementary Material).

### 3.3 Training and Evaluation

All models were trained and evaluated on the same cohort of 202 patient-specific AAA graphs using a consistent sampling, augmentation, and cross-validation strategy. To avoid information leakage and preserve the distribution of thrombus burden across splits, we performed patient-level stratified splitting based on ILT class. Thus, for each fold, 80% of the cases were assigned to the training set and 20% to the test set, with low-, moderate-, and high-ILT aneurysms, defined according to the ratio of ILT volume to AAA sac volume, proportionally represented in both subsets. This procedure was repeated for $N_{\text{fold}}=5$ different random seeds, yielding five independent 80/20 train–test partitions per architecture; all reported metrics were averaged over the five folds.

Within each training split, the graphs were standardized using feature scalers computed from the training cases only. Node-specific geometric/biomechanical descriptors, graph-level attributes, coordinates, and wall stresses were z-scored. To improve robustness and reduce overfitting in this cohort, we applied the same data augmentation and sampling scheme for the three model types. At the graph level, we perturbed selected global features (e.g., ILT burden measures) with small multiplicative Gaussian noise and clipped them to physiologically plausible ranges. At the node level, we injected feature-wise noise into key ILTTHK, curvatures, and centerline descriptors and enforced non-negativity or upper bounds where appropriate. In addition, we performed stress-stratified subsampling within each graph during training: nodes were partitioned into low, medium, and high stress bins based on the FEA-estimated values, and a fixed fraction of each bin was retained so that high stress regions were consistently represented despite being infrequent on the surface. Node-specific loss terms were weighted with stress-based factors to further emphasize the upper tail of the wall stress distribution. For each fold and model, the training optimized a composite objective on normalized wall stresses $\left\{{\overset{ˆ}{\sigma }}_{i}\right\}$ using Adam optimizer with weight decay and a ReduceLROnPlateau scheduler. The loss combined a wall stress-weighted node-specific mean squared error with penalties on discrepancies between predicted and true $\overset{‾}{\sigma }$ and between predicted and true $\sigma_{99}$, plus a pinball (quantile) term at the 0.99 level to explicitly constrain the high wall stress tail. Training proceeded for up to 1500 epochs with early stopping based on the training loss, while the best-performing weights (per fold) were checkpointed together with the corresponding feature scalers.

The evaluation began at the node level on the test data set graphs for each fold. We computed the root mean squared error (RMSE), relative error (RE), and coefficient of determination $R^{2}$ between the denormalized predictions ${\overset{ˆ}{\sigma }}_{i}$ and the FEA reference $\sigma_{i}$. In addition, we calculated the spatial agreement measured by the Pearson correlation between predicted and simulated stress fields on each AAA wall surface. Graph-level quantities were then derived directly from these node-specific fields: for every AAA we computed the $\overset{‾}{\sigma }$ and the $\sigma_{99}$ using the predicted and ground truth FEA distributions, and reported RMSE, RE and $R^{2}$ for the test cohort. This formulation tested whether the models preserve clinically significant global load descriptors, particularly the high stress tail represented by $\sigma_{99}$, even when perfect agreement at every node is not feasible. All random seeds, stratified splits, feature-scaling parameters, and best-performing model weights (per fold and architecture) were archived, and training hyperparameters and hardware details are summarized in Table B1 of the Supplementary Material to allow exact replication of the numerical experiments.

## 4 Results

For the best-performing stratified split, which is used for all exemplar case visualizations and qualitative analyses, the three architectures accurately reconstructed the node-specific wall stress field (${\overset{ˆ}{\sigma }}_{i}$) for the test data set, with node-specific $R^{2}$ values exceeding ~ 0.97 and graph-level $R^{2}$ for $\sigma_{99}$ exceeding ~ 0.98. For nodes with $\sigma_{i}\geq 5\;\text{N}/{\text{cm}}^{2}$, typical relative errors are in the ~ 12–20% range, with the GT consistently achieving the lowest median RE and lower upper-tail errors than GGCN and EGNN. The highest absolute and relative errors are concentrated in the highest stress nodes, where the true stresses are substantially greater than the mean; thus, increases in absolute error reflect the greater magnitude of $\sigma_{i}$ rather than a loss of relative accuracy of the model. Among the three, GT achieved the highest node-specific stress and $\sigma_{99}$ accuracies, followed by GGCN and EGNN, indicating that attention-based aggregation on the aneurysm mesh is particularly effective in this setting.

### 4.1 GNN-predicted Wall Stress and Comparison with Ground Truth

**Figure 4.**
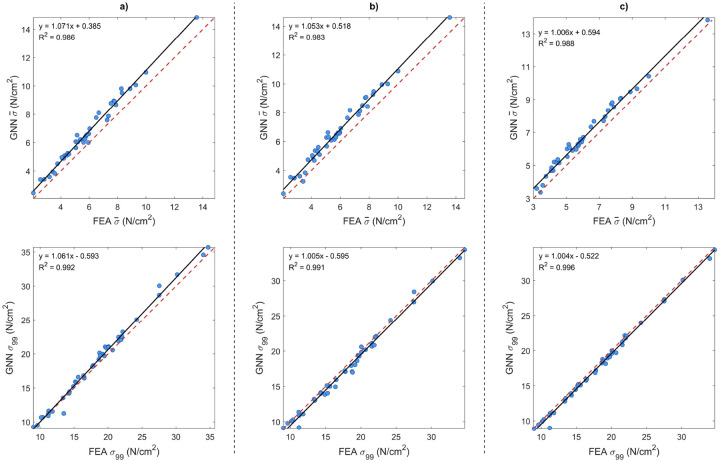
Test data set agreement between GNN-predicted and FEA-derived $\overset{‾}{\sigma }$ and $\sigma_{99}$ for the best stratified fold for a) GGCN, b) EGNN, and c) GT. Solid black lines indicate least-squares fits; dashed red lines denote the identity line.

Graph-level stress metrics derived from node-specific predictions also showed excellent agreement with the FEA ground truth on the best-fold test set. Figure 4 summarizes the comparisons of GNN-predicted vs. FEA-derived $\overset{‾}{\sigma }$ and $\sigma_{99}$ for each GNN architecture per AAA in the test data set. All three models lie close to the identity line with regression slopes near unity and $R^{2}$ greater than ~ 0.97, but the GT consistently yields the smallest scatter and deviations from the identity line, particularly for the high stress tail quantified by $\sigma_{99}$. For individual AAAs in this fold, relative errors in graph-level metrics remained modest: for most cases, RE for $\overset{‾}{\sigma }$ was less than 15% and less than 10% for $\sigma_{99}$, with GT yielding the lowest median and upper-tail relative errors. GGCN closely follows the GT performance, while EGNN exhibits a slightly larger spread for $\sigma_{99}$, which is consistent with its lower node-specific $R^{2}$.

**Figure 5.**
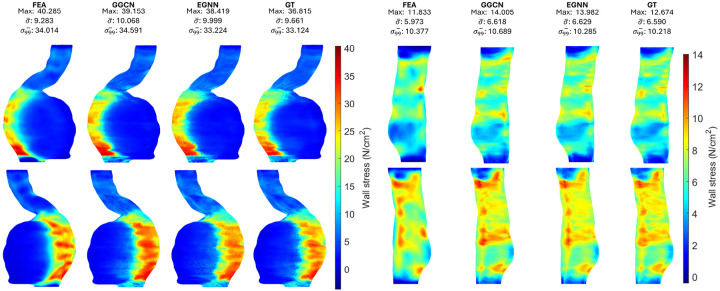
Spatial agreement of wall stress fields for two exemplary AAAs from the best-fold test set, one with relatively high (left frame) and one with relatively low (right frame) wall stresses. Rows show two opposite views of the same AAA, and columns show FEA ground truth, GGCN, EGNN, and GT predictions; color maps correspond to the first principal wall stress distributions projected onto the outer wall surface.

**Figure 6.**
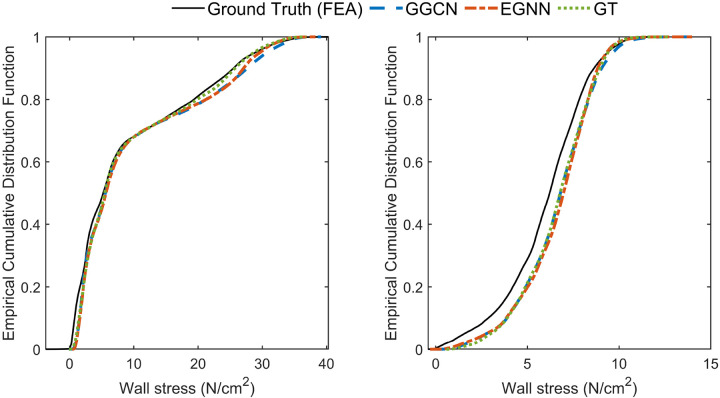
Empirical cumulative distribution functions (ECDFs) for the same AAAs shown in Figure 5. The left plot corresponds to the AAA illustrated on the left frame of Figure 5, while the right plot corresponds to the AAA illustrated on the right frame of Figure 5.

To examine spatial fidelity in detail, we selected two exemplary AAAs from the test set with high FEA $\sigma_{99}$ and complex geometry. Figure 5 shows the predicted and reference stress maps for a representative case, and Figure 6 displays the corresponding empirical cumulative distribution functions (ECDFs) of node-specific stresses. For all GNNs, high stress regions near the sac shoulder and other regions of high curvature are accurately localized; the GT provides the closest match to the FEA maps and distributions, followed closely by the GGCN, while the EGNN tends to slightly smooth the highest peaks while preserving their locations and relative magnitudes.

### 4.2 Cross-Validation Results and Performance Evaluation

To rigorously evaluate model generalization and prevent overfitting, we employed a 5-fold stratified cross-validation approach in our data set of 202 patient-specific AAA geometries. The stratification was performed according to the ILT classification to ensure a balanced representation of different anatomical variants between training and test sets. The metrics reported for each fold were obtained solely from the corresponding 41 test cases of that fold, ensuring that none of these test cases were used in the training set of the same fold.

#### 4.2.1 Cross-validation performance and variability

Table 1 summarizes the performance of the models for the 5-fold cross-validation in terms of RMSE, RE, and $R^{2}$. Although the three architectures achieve high $R^{2}$ values for both $\overset{‾}{\sigma }$ and $\sigma_{99}$, GT consistently achieves the lowest RMSE and RE, particularly for $\sigma_{99}$, indicating a more accurate recovery of the high stress tail in physical units (N/cm^2^). GGCN and EGNN exhibit similar $R^{2}$ values compared to GT, but their higher RMSE and RE reveal systematically larger discrepancies in predicted stress magnitudes, even when the variance explained is comparable.

#### 4.2.2 Individual fold analysis

Table 2 complements the aggregate metrics in Table 1 by reporting RMSE for each cross-validation fold. For $\overset{‾}{\sigma }$, GT consistently achieves the lowest RMSE in four out of the five folds, with a mean RMSE of 0.671 N/cm^2^ compared to 0.741 N/cm^2^ for GGCN and 0.775 N/cm^2^ for EGNN. For $\sigma_{99}$, all models incur larger errors due to the higher variance of this tail metric. However, GT exhibits the lowest mean RMSE averaged for all folds.

## 5 Discussion

The present work demonstrates the feasibility of using GNN architectures to predict patient-specific AAA wall stress distributions directly from geometric descriptors extracted from CTA-derived surface meshes of the outer wall. For the three model types, namely GGCN, EGNN, and a local GT, the node-specific wall stress field and graph-level 99th percentile wall stress were reconstructed with high fidelity relative to the FEA-derived ground truth. Evaluated on a test data set, the proposed GNN surrogates reproduced wall stresses calculated with FEA with high accuracy while reducing the inference time from hours to seconds, transforming patient-specific wall stress mapping from an offline research tool into a potentially real-time component of clinical decision support.

### 5.1 Key Findings and Clinical Implications

All three GNN architectures achieved on average $R^{2}>0.970$ for $\sigma_{99}$, with GT providing the best overall trade-off between accuracy and stability and the lowest $\sigma_{99}$ RE (0.073). It should be noted that a $\sigma_{99}$ RE in the interval 0.07–0.10 represents a small fraction of the typical wall stress for an asymptomatic AAA (~5–30 N/cm^2^) and therefore is likely to be clinically acceptable for risk stratification. Performance was stable across the folds: the standard deviation of $R^{2}$ for $\overset{‾}{\sigma }$ and $\sigma_{99}$ was in the range 0.01–0.02 for all models, with GT showing particularly low variability ($\overset{‾}{\sigma }:R^{2}=0.979\pm 0.018;\sigma_{99}:R^{2}=0.985\pm 0.012$).

Even in the most challenging folds, all models maintained high $R^{2}$ values for $\overset{‾}{\sigma }$ and $\sigma_{99}$, suggesting that wall stress for complex AAA geometries can be reproduced with good fidelity. Finally, by matching the FEA ground truth while reducing the wall stress inference times to seconds, these GNN-based surrogates have the potential to support near real-time clinical decision making in settings where FEA modeling may be impractical or where existing biomechanical tools (e.g., A4 Clinics) are limited by computation time and workflow complexity^16, 17, 47^.

### 5.2 Model Performance, Spatial Agreement, and Error Estimates

Qualitative and quantitative analyses showed that all GNNs achieved high node-specific predictive accuracy and strong agreement for the estimates of $\overset{‾}{\sigma }$ and $\sigma_{99}$. Wall stress maps for exemplary AAAs demonstrated accurate localization of high wall stress regions near the sac shoulder and other areas of high curvature, with GT providing the closest match to the FEA ground truth. These findings indicate that incorporating curvature-based features (e.g., $k_{1},k_{2},K,M$), WALLTHK, and ILTTHK provide a biomechanically meaningful context that the models can exploit to reconstruct spatial stress patterns. This is consistent with previous work associating curvature, wall thickness, and ILT burden with AAA wall stress and rupture risk^24, 48^.

The structure of node-specific errors further supports the reliability of the surrogates. The error distributions were narrowly centered around zero with only mild skewness, and most of the nodes exhibited stresses ± 2 N/cm^2^ of the FEA ground truth even in high stress regions. At the graph level, the three architectures tend to slightly overestimate $\overset{‾}{\sigma }$; GGCN shows a small positive bias for $\sigma_{99}$, while EGNN and GT are nearly unbiased for $\sigma_{99}$. Although variance increased concomitantly with wall stress and all models were less accurate at the highest stresses, the errors were modest: near $\sigma_{99}$, typical absolute deviations (RMSE) were in the range of 1–2 N/cm^2^. These absolute deviations correspond to RE less than 0.10, in line with the cross-validation results in Table 1. GT consistently exhibited the smallest error distribution and smallest high magnitude tails, followed by GGCN and EGNN, underscoring its robustness in the clinically critical high stress regime. We infer from this that GT’s attention-based aggregation on the aneurysm wall surface provides a flexible mechanism to capture long-range geometric interactions that are not easily encoded by purely local message passing. In contrast, EGNN’s explicit equivariance to rigid motions, while attractive from a geometric learning perspective, did not translate into superior accuracy. This is likely due to the fact that key geometric information is adequately represented by the curvature and thickness descriptors and because strict equivariance may limit representational flexibility on noisy, patient-specific surface meshes.

### 5.3 Importance of High Stress Region Sensitivity

Accurate identification of high stress regions is critical for rupture risk assessment, as AAA failure is expected to initiate where wall stress exceeds the local wall strength rather than where the mean stress is highest. Pure MSE training tends to underestimate the high stress regions due to the dominance of low- and moderate-stress nodes in the loss. The composite objective used in this work, which combines a stress-weighted node-specific MSE with penalties on $\overset{‾}{\sigma },\sigma_{99}$, and a high-quantile pinball loss, improved accuracy in the stress tail and reduced systematic underestimation of $\sigma_{99}$, as reflected in its narrow error distributions. This behavior is consistent with previous work in medical image regression^49^ and quantile regression^50^, where targeted loss terms improve performance on clinically important extremes and support the use of tailored objectives when high-risk regions drive clinical decisions. It is important to note that $\sigma_{99}$ alone is an incomplete surrogate of rupture risk, as $\sigma_{\text{ult}}$ and its spatial heterogeneity are also necessary to adequately estimate risk^17, 51^. Several studies have reported that peak wall rupture index, which combines local stress and wall strength, provides better discrimination between ruptured and asymptomatic AAAs than peak wall stress alone. In this context, the present framework should be viewed as a fast geometry-to-stress module that can be coupled with statistical or image-based wall strength models to estimate the peak wall rupture index.

### 5.4 Advantages over Finite Element Modeling

Compared with conventional FEA modeling pipelines, the GNN-based surrogates offer several practical advantages. Once trained, they can predict the full AAA wall stress field in seconds, while FEA simulations typically require hours and expert supervision for mesh preparation, boundary-condition specification, and material calibration^47^. The proposed approach requires the segmented outer wall, inner wall, and lumen surfaces with derived geometric descriptors, and can therefore be more readily integrated into semi-automated imaging workflows. This computational efficiency and reduced pre-processing burden would make patient-specific wall stress assessment feasible in a clinical environment, echoing emerging efforts to employ deep neural networks as fast surrogates for vascular FEA modeling^52^. In addition, the proposed GNN framework is suitable for repeated or longitudinal analyses, where rapid recomputation of wall stress fields for evolving aneurysm geometries could support closer surveillance of biomechanical risk than what is currently possible with FEA. Fast surrogates may also enable probabilistic analyses, such as Monte Carlo sampling of material properties or blood pressure, which are often prohibitively expensive with traditional simulations but are relevant for calculating uncertainty in rupture risk estimates.

### 5.5 Limitations, Generalizability, and Future Work

This work is subject to several important limitations. The GNN models were trained and evaluated on a data set of 202 patient-specific geometries generated from CTA exams obtained at three clinical centers. A broader validation on larger, multi-center cohorts with more heterogeneous imaging protocols will be needed to confirm generalizability of the GNN predictions. In addition, the FEA ground truth assumed homogeneous isotropic wall and ILT material properties, whereas in vivo tissue properties are believed to be spatially heterogeneous. In addition, patient-specific material properties are never known *a priori*, which should be relevant for FEA modeling considering that ILT can substantially modulate local wall stress^1, 16^. These modeling simplifications are shared with most prior biomechanical AAA studies, but may limit the absolute accuracy of the predicted wall stresses and hence of any subsequent rupture risk metrics. Future work should focus on extending the data set and systematically exploring alternative constitutive material model assumptions for the FEA ground truth, including anisotropic or spatially heterogeneous wall and ILT material models. Furthermore, augmenting the GNN with uncertainty quantification and calibration could provide case-wise confidence intervals for $\overset{‾}{\sigma }$ and $\sigma_{99}$, which would further support clinical translation and shared decision-making for elective AAA repair. Another promising direction is to integrate the current geometry-based surrogate into a broader *digital twin* framework that combines wall stress with patient-specific hemodynamics, inflammatory biomarkers, and peak wall rupture index to provide a more comprehensive biomechanical risk stratification strategy^51^.

## 6 Conclusion

We developed, trained and tested geometry-aware graph neural network surrogates to predict patient-specific AAA wall stress distributions directly from CTA-derived surface meshes. Using a cohort of 202 AAA geometries, the three GNN architectures (GGCN, EGNN, and a local GT) reproduced FEA-estimated wall stress fields, $\overset{‾}{\sigma }$ and $\sigma_{99}$ with high fidelity while reducing inference times from hours to seconds per AAA. The local GT consistently delivered the most accurate and stable predictions, particularly in the clinically critical high stress regions, suggesting that attention-based aggregation on curvature- and thickness-enriched meshes is well suited for the overall goal of the framework. These findings support the use of fast GNN-based surrogates as a key building block for AAA *digital twins* and motivate future integration with patient-specific material modeling, uncertainty quantification, and rupture risk assessment as part of a collective strategy for the clinical management of AAAs under surveillance.

## Supplementary Material

Supplementary Files

This is a list of supplementary files associated with this preprint. Click to download.

- SupplementaryMaterialCarvajaletal.pdf

## Figures and Tables

**Table 1.** Summary of model performance across 5-fold cross-validation (mean ± standard deviation). RMSE is reported in N/cm^2^; RE is the mean relative error calculated for all the test cases.

Model	Metric	RMSE (N/cm^2^)	RE	$R^{2}$
GGCN	$\bar{\sigma }$	0.741 ± 0.086	0.093 ± 0.021	0.977 ± 0.013
	$\sigma_{99}$	1.054 ± 0.345	0.082 ± 0.034	0.979 ± 0.015
EGNN	$\bar{\sigma }$	0.775 ± 0.066	0.098 ± 0.020	0.974 ± 0.016
	$\sigma_{99}$	1.019 ± 0.270	0.087 ± 0.030	0.984 ± 0.010
GT	$\bar{\sigma }$	0.671 ± 0.059	0.082 ± 0.016	0.978 ± 0.016
	$\sigma_{99}$	0.926 ± 0.371	0.073 ± 0.035	0.984 ± 0.011

**Table 2.** RMSE (in N/cm^2^) for $\overset{‾}{\sigma }$ and $\sigma_{99}$ for the five folds of the cross-validation test for each GNN model.

Model	Metric	Fold 1	Fold 2	Fold 3	Fold 4	Fold 5
GGCN	$\overset{‾}{\sigma }$	0.673	0.794	0.747	0.625	0.867
	$\sigma_{99}$	1.448	1.079	1.368	0.498	0.879
EGNN	$\overset{‾}{\sigma }$	0.704	0.787	0.764	0.729	0.893
	$\sigma_{99}$	1.224	1.053	1.383	0.660	0.776
GT	$\overset{‾}{\sigma }$	0.636	0.784	0.642	0.618	0.675
	$\sigma_{99}$	1.296	0.998	1.334	0.406	0.594

## Data Availability

The data set used in this study is not publicly accessible due to IRB regulations in the respective clinical centers. However, it can be obtained by making a reasonable request to the corresponding author.
